# Transition care: the link between pediatric rheumatology and adult rheumatology. Data from the experience of the young adult rheumatology ambulatory of Florence

**DOI:** 10.1186/1546-0096-12-S1-P11

**Published:** 2014-09-17

**Authors:** Fernanda Falcini, Federico Bertini, Gemma Lepri, Alessandra Radicati, Marco Matucci Cerinic

**Affiliations:** 1 Department of BioMedicine, Section of Rheumatology, Transition Clinic, University of Florence, Florence, Italy

## Introduction

Transition from childhood to adolescence is particularly difficult in patients with chronic rheumatic diseases. Moving from pediatric to adult assistance, is not an administrative procedure; it involves a change from of "child-centered" to an "adult-oriented" system, in a delicate phase of life in which emotional stability has not been achieved yet. A structure addressing medical, psycho-social and educational adolescents needs is therefore necessary, representing a link between adult and pediatric rheumatology, in order to maintain the benefits of treatment administered during in childhood.

## Objectives

We report the experience of Young Adults Rheumatology Outpatient of Florence taking charge of the patients in this phase.

## Methods

From January 2000 to May 2014, 754 patients were visited at the Transition Outpatient: 41% with JIA; 16% arthralgia; 9% Raynaud's phenomenon; 6% SLE; 3% scleroderma; 2% connective tissue; 2% dermatomyositis; Acute Rheumatic Fever (ARF) 2%; 1% autoinflammatory syndromes; 18% others. Of the 311 patients with JIA (93M, 218F), 58.8% had oligoarticular onset; 21.2% polyarticular; ERA 13.8%; 4.5% psoriatic arthritis and 1.6% systemic onset. Uveitis was found in 29/311 patients, of which 76% with oligoarthritis.

## Results

147 patients, all with JIA, were evaluated for the involvement of the temporomandibular joint that was noted in 73/147; of these, 29/73 are affected by oligoarticular JIA, 18/73 polyarticular, 15/73 ERA, 7/73 psoriatic and 4/73 systemic onset. 66/311 (21.2%) patients with JIA are treated with biologics: 32/66 etanercept, 27/66 adalimumab, 3 abatacept, 3 tocilizumab and 1 golimumab. The prevalence of female gender is observed among patients with Raynaud’s (76%) and SLE (91%). Of the 25 patients with scleroderma, 22 (88%) had localized form, 3 (12%) the systemic one. Out of the 13 patients with ARF (9M, 4F), 9 (69%) had cardiac involvement. Among the 134 patients defined as "others": 7 have Kawasaki Disease, 3 Takayasu and 3 PAN.

## Conclusion

Our experience shows that over than 60% of JIA patients had active disease despite biological therapy; all SLE patients had active disease in varying degrees and require therapy. Osteopenia/osteoporosis, chronic uveitis, alterations in the TMJ required ongoing specialty care. Contraception and pregnancy should be handled with special care, in particular in SLE. It is crucial to work closely with other specialists, common in our center. It is necessary to assiduously support, understand and care of these young patients to prevent therapy suspension, as they frequently want to achieve independence from drugs and become equal to healthy peers. It would desirable: 1) to achieve a better collaboration between pediatricians and adult rheumatologists for a gradual transition between the two types of assistance; 2) to organize Transition Outpatients Clinics to make this transition less dramatic and maintain adherence to therapy and disease control.

## Disclosure of interest

None declared.

